# The Double-Edged Sword: How Radiotherapy Shapes the Tumor Immune Microenvironment to Modulate Responses to Checkpoint Inhibitors

**DOI:** 10.3390/ijms27104525

**Published:** 2026-05-18

**Authors:** Chen-Hsuan Chiang, Hui-Wen Chan, Hui-Yen Chuang

**Affiliations:** Department of Biomedical Imaging and Radiological Sciences, National Yang Ming Chiao Tung University, Yangming Campus, No. 155, Sec. 2, Li-Nong St., Beitou, Taipei 112, Taiwan

**Keywords:** radiotherapy, tumor microenvironment, immune checkpoint inhibitors, immunomodulation, cGAS-STING, ferroptosis, tumor metabolism

## Abstract

Radiotherapy (RT) is a cornerstone of cancer treatment, traditionally recognized for its direct cytotoxic effects via DNA damage. However, emerging evidence highlights RT as a profound modulator of the tumor microenvironment (TME), acting as a “double-edged sword” that greatly influences the success of immune checkpoint inhibitors (ICIs). On the one hand, RT acts like an in situ vaccine, causing immunogenic cell death and activating the cGAS-STING pathway, which leads to dendritic cell maturation, T-cell infiltration, and reactive PD-L1 expression. This effect can turn “cold” tumors into “hot” ones, making them more responsive to immune checkpoint blockade. On the other hand, RT can lead to resistance to ICIs by promoting an immunosuppressive environment, recruiting regulatory T cells, M2 macrophages, and myeloid-derived suppressor cells. This review analyzes the mechanisms behind this immunological duality and assesses how parameters such as dose, fractionation, and particle type (e.g., carbon ion versus photon therapy) can be optimized to enhance immune activation. Lastly, we discuss future strategies that focus on innate immunity and tumor metabolism, showing how targeting nutrient depletion and ferroptosis can break down immunosuppressive barriers and position RT as an essential component of precision immuno-oncology.

## 1. Introduction

Radiotherapy (RT) is a key modality in cancer treatment, as more than half of all cancer patients receive RT for various therapeutic purposes. For cancer treatment, RT works by applying ionizing radiation to cause DNA damage either directly or indirectly, ultimately leading to cell death [1]. Direct effects occur when radiation deposits energy directly into DNA, causing irreparable damage and cell death. Indirect effects happen when radiation transfers its energy to nearby water molecules, creating reactive oxygen species (ROS) that cause widespread cellular damage through various mechanisms.

Beyond inducing local cytotoxic effects, RT can also trigger responses outside the irradiated area, leading to two remarkable phenomena called the bystander effect and the abscopal effect. The bystander effect describes damage to adjacent unirradiated cells via short-range signals emitted by irradiated cells [2], while the abscopal effect refers to a systemic, out-of-field response that is now understood to be immune-mediated [3]. Recently, the emergence of immune checkpoint inhibitors (ICIs), such as anti-PD-1/PD-L1 and anti-CTLA-4 therapies, has transformed clinical oncology and treatment approaches. Nonetheless, ICIs often fail to produce responses in non-immunogenic “cold” tumors that lack existing tumor-infiltrating T cells. As noted, RT can activate systemic immune responses and enhance the effectiveness of ICIs by converting “cold” tumors into “hot” ones, increasing T-cell infiltration [4].

However, it is worth noting that RT-mediated immunological impact is a double-edged sword. On the one hand, RT can initiate powerful anti-tumor immune responses by enhancing the release of damage-associated molecular patterns (DAMPs) and tumor antigens, promoting the recruitment of immune cells, and activating systemic anti-tumor immune responses. Through this process, RT can successfully reshape the TME into an immunogenic “hot” state with robust antigen-specific T-cell infiltration, making irradiated tumors more susceptible to ICI treatments [5,6]. On the other hand, RT can also act as a selective pressure, fostering a pro-tumor immunosuppressive TME by recruiting and enriching immune-inhibitory cells, such as regulatory T cells (Tregs), M2 macrophages, and myeloid-derived suppressor cells (MDSCs). Furthermore, RT-induced inflammatory signaling often paradoxically upregulates immune checkpoint expression on residual tumor cells, driving immune evasion and treatment resistance [7,8].

Understanding this duality is critical for designing rational strategies that maximize the synergistic benefits of combined RT and immunotherapy. This manuscript explores the complex mechanisms by which RT modulates the TME, thereby determining the success or failure of immune checkpoint blockade. By thoroughly analyzing how various radiation parameters, such as radiation types and fraction sizes, affect RT-mediated immune modulation, and by highlighting future directions, including targeting tumor metabolism and boosting ferroptosis or cuproptosis, we aim to offer a practical approach to overcoming acquired ICI resistance seen in current cancer therapy.

## 2. The Anti-Tumor Edge: How Radiation Activates Immunity

RT can act as an in situ vaccine, triggering a series of events that convert the non-immunogenic “cold” TME into an immunogenic “hot” TME, thereby enhancing systemic anti-tumor immunity and responsiveness to immune checkpoint inhibitors. The overall process begins with RT-induced cell death and is followed by the mobilization and activation of immune cells, as described below.

### 2.1. Mechanism of Immunogenic Cell Death (ICD) and CTLA-4 Priming

**Figure 1:**
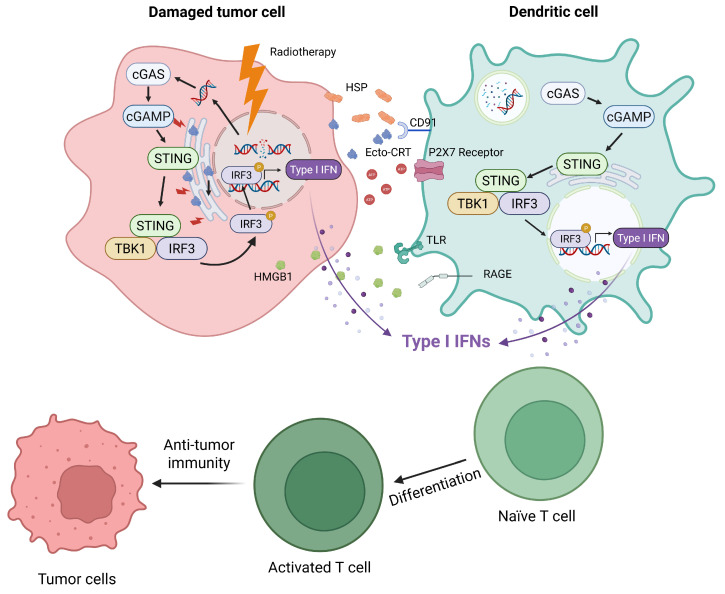
A schematic illustration of RT-induced ICD and subsequent anti-tumor immune activation. RT induces DNA damage and ER stress in tumor cells, leading to the exposure of DAMPs, such as ecto-CRT, ATP, HSPs, and HMGB1. These signals activate DCs through CD91, P2X7, TLRs, and RAGE, promoting DC maturation and antigen presentation. In parallel, cytosolic DNA fragments trigger the cGAS–STING pathway, resulting in type I interferon production and recruitment of DCs. Activated DCs cross-prime tumor antigens to CD8^+^ T cells, which then mediate anti-tumor response. Created in Biorender. Chan, H.-W. (2026) https://BioRender.com/z7klkzg.

The type of cell death caused by RT is a critical determinant of the subsequent immune response [5]. Cell death generally falls into two categories: non-immunogenic and immunogenic cell death (ICD) (Figure 1). For instance, apoptosis is often considered non-immunogenic or “silent,” although tumor-associated antigens (TAAs) can be released during this process [6]. Nonetheless, antigen-presenting cells (APCs), like dendritic cells (DCs), often fail to fully mature because they receive insufficient DAMPs. It is known that co-stimulatory signals, such as CD28, expressed by mature APCs, are required for successful T cell activation. T cells may become anergic (unresponsive) or undergo activation-induced cell death (AICD) when encountering their designated antigens, a process often mediated by TNF-related apoptosis-inducing ligand (TRAIL) signaling [7]. Without enough co-stimulatory signals from these immature APCs, naïve T-cell priming becomes ineffective, resulting in weak anti-tumor immunity. Conversely, ICD induced by RT effectively stimulates strong anti-tumor immune responses, mainly by releasing DAMPs that serve as immunostimulatory signals and triggering APC maturation [8].

After RT, key DAMPs, including calreticulin (CRT), ATP, heat shock proteins (HSPs), and high-mobility group box 1 (HMGB1), are released. In damaged cells, surface-expressed ecto-calreticulin (ecto-CRT) is an early ICD indicator following RT [8]. RT-induced ER stress causes CRT to move to the cell surface, where it binds to CD91 on DCs, promoting phagocytosis, sending immunostimulatory signals [9], and triggering DC activation [10]. Moreover, a correlation between decreased ecto-CRT and reduced DCs in the draining lymph nodes has been reported [11]. Extracellular ATP (eATP) also drives DC activation by acting as a “find-me” signal and binding to the P2 × 7 receptor, thereby enhancing antigen uptake and promoting maturation. Mature DCs then release pro-inflammatory cytokines like IL-1β to support CD8^+^ T cell activation and differentiation [12]. Heat shock proteins (HSPs), another DAMP type, are transported to the cell surface or secreted, serving as danger signals. They are recognized by APCs through CD91 or Toll-like receptors (TLRs), triggering anti-tumor immune responses [13].

Lastly, HMGB1 is a DAMP released during the late stage of cell death when the cell membrane loses its integrity. It binds to receptors such as TLR4 on DCs, prompting cytokine production, enhancing antigen presentation, and aiding the priming of tumor-specific CD8^+^ T cells. This robust maturation of DCs provides the essential CD80/CD86 co-stimulatory signals that influence T cell differentiation by interacting with CTLA-4 [14]. Blocking CTLA-4 during this critical phase can boost priming effectiveness in an anti-cancer vaccine in a cancer model [15] and may enhance the effects of RT-induced antigen presentation.

### 2.2. The cGAS-STING Pathway: Driving PD-L1 Expression and Antigen Presentation

The efficacy of RT extends beyond direct cytotoxicity; it fundamentally reshapes the immunological landscape within tumors. RT-induced DNA damage initiates an intracellular innate immune response, primarily through the cGAS-STING pathway, which is activated when DNA fragments are released into the cytosol [15]. This activation transforms local cellular damage into a systemic innate immune response by driving the production of type I interferons, which are essential for recruiting and maturing DCs. Matured DCs then cross-present tumor antigens to cytotoxic CD8^+^ T cells, leading to the following antigen-specific cell killing [16]. Importantly, this interferon signaling simultaneously drives the reactive expression of PD-L1 on both tumor cells and infiltrating immune cells [17,18]. This adaptive upregulation of PD-L1 acts as a localized immunosuppressive shield and a mechanism of radioresistance [19,20]. RT-induced PD-L1/L2 upregulation perfectly sets the stage for anti-PD-1/PD-L1 inhibitors [17,18], providing the precise molecular targets required for these drugs to unleash cytotoxic CD8^+^ T-cell responses. The Phase III PACIFIC trial exemplifies this mechanistic synergy, establishing durvalumab as the standard care after chemoradiotherapy for unresectable stage III non-small cell lung cancer (NSCLC), demonstrating RT-induced TME modifications can be effectively targeted by checkpoint blockade [19].

Specific recognition of cancer cells (i.e., tumor-specific and tumor-associated antigens) significantly affects the cytotoxic CD8^+^ T-cell response. Many cancer cells evade immune surveillance by reducing major histocompatibility complex class I (MHC-I) expression, effectively making themselves invisible. Interestingly, RT has been shown to dose-dependently increase MHC-I levels in irradiated cells. In addition to increasing MHC-I molecules, RT also alters the immunopeptidome of cancer cells, generating unique neoantigens induced by RT that could serve as new targets for T cells [20,21]. In other words, combining RT with checkpoint blockade can enhance T-cell-mediated cytotoxicity by activating cytotoxic CD8^+^ T cells and increasing the number of neoantigens targeted for attack.

For instance, fractionated RT has been demonstrated to enhance T-cell susceptibility in poorly immunogenic 4T1 cells by increasing the mutation rate, neoantigen expression, and levels of death receptors such as FAS/CD95 and DR5 [22]. CD8^+^ T-cell function is regulated by CD4^+^ T cells and suppressed by immunosuppressive CD4^+^/CD25^+^/Foxp3^+^ Tregs. The immunosuppressive and immunomodulatory roles of Tregs have been demonstrated by combining anti-CD25 antibody with RT, thereby strengthening the RT-induced abscopal effect [23]. Interestingly, RT has been shown to reduce Foxp3 expression in induced Tregs (iTregs) without additional agents, thereby lowering their capacity to suppress CD8^+^ T-cell proliferation [24].

## 3. Radiation-Induced Reprogramming of the Tumor Immune Microenvironment to Augment ICI Efficacy

### 3.1. Reprogramming the TME to Overcome ICI Resistance

In parallel with its direct effects on cancer cells, RT also reprograms immune cells within the TME, thereby shaping the balance between anti-tumor immunity and immune suppression. This reprogramming involves a cascade of events, from the initial priming of the immune system to the conversion of the tumor landscape and synergy with advanced immunotherapies, particularly immune checkpoint inhibitors (ICIs).

### 3.2. Initiating the Anti-Tumor Response: DC Maturation and T-Cell Priming

An effective antigen presentation is the first step in generating a strong anti-tumor immune response. In addition to inducing effective cell death, RT excels at activating DCs through ICD, then initiating an anti-tumor immune response. High-dose (10 Gy) RT has been reported to induce robust ICD, characterized by increased ecto-CRT, HMGB1, and HSPs exposure. Upregulation of these DAMPS drives substantial DC maturation, characterized by increased expression of co-stimulatory molecules, such as MHC-II, CD80, and CD86, and the production of pro-inflammatory cytokines, like IL-12 and TNF-α [25]. This increased activation state enables DCs to effectively prime tumor-specific cytotoxic CD8^+^ T cells and support CD4^+^ T helper responses. Combining RT with DC-targeted adjuvants can significantly enhance these processes, helping overcome both pre-existing and acquired resistance to ICIs, such as anti-PD-L1 [25], by promoting DC maturation and subsequent T-cell priming. As mentioned, DCs play crucial roles in initiating anti-tumor responses; however, recent research suggests that the timing of irradiation may also be important for achieving positive results. Telarovic and colleagues showed that delayed irradiation to the tumor-draining lymph node (TDLN) can significantly enhance ICI efficacy by preserving DC homing ability through the CCR7–CCL19/CCL21 chemokine pathway. This study convincingly demonstrated how the RT schedule can affect overall outcomes when combined with ICIs, particularly concerning DC migration, antigen presentation, and T-cell priming [26].

### 3.3. Enhancing the Effector Response: Converting the TME for ICI Success

In addition to boosting T-cell priming, RT promotes T-cell infiltration and activity within tumors, shifting the TME from an immune “cold” to a “hot” state, which is essential for the effectiveness of ICI. Both high-dose [27] and low-dose [28] RT have been shown to increase T-cell infiltration into tumors and enhance treatment outcomes in lung and gastric cancer models, although the underlying mechanisms may differ. Since RT can promote T-cell infiltration, combining CAR-T therapy with RT has been suggested and demonstrated to yield positive results, even in eradicating contralateral tumors, suggesting an enhanced abscopal effect [29].

RT directly triggers the activation and expansion of resident stem-like CD8^+^ T cells, which then differentiate into functional cytotoxic CD8^+^ T cells. This mechanism was confirmed experimentally by using FTY720 to block the infiltration of new lymphocytes. Surprisingly, RT still improved tumor control under these conditions, showing its ability to activate the pre-existing T cell population [30]. The cytotoxic potential of these RT-activated T cells could be further unleashed when combining RT with ICIs, such as anti-PD-1/PD-L1 treatment.

Low-dose radiotherapy (LDRT) has been introduced as a new approach to modify the TME. LDRT not only enhances DC maturation but also promotes the infiltration of activated DCs and reprograms both the innate and adaptive immune systems to boost anti-tumor immunity. This idea has been validated in a clinical trial (NCT03728179), where LDRT led to increased T-cell infiltration in patients with previously non-responsive tumors, significantly improving their response to subsequent immunotherapy [31].

It is widely recognized that TAMs are the most abundant innate immune cells within the TME [32]. Based on the TME cues, TAMs can polarize into a pro-inflammatory M1-like phenotype that enhances anti-tumor immunity or an anti-inflammatory M2-like phenotype that facilitates tissue repair, angiogenesis, and immune suppression [33]. RT can shift the balance of TAMs toward a pro-inflammatory, anti-tumor M1-like phenotype. This beneficial reprogramming is evidenced by increased pro-inflammatory markers in macrophages [34] and a favorable increase in the intra-tumoral M1/M2 ratio [35].

Targeting macrophage function is a crucial strategy to bolster the anti-tumor effectiveness of RT. Inhibiting histone deacetylase 6 (HDAC6) can maintain macrophages in a pro-inflammatory state, boosting anti-tumor immunity [36]. The combination of the HDAC6 inhibitor SP-2-225 and RT has been shown to increase the M1/M2 ratio and expand various CD8^+^ T cell populations, including central and effector memory T cells. This combination not only strengthens anti-tumor responses but also reduces tumor recurrence after RT by promoting the development of memory T cells [37]. It establishes an optimal inflammatory environment for future combination therapies with ICIs.

As innate lymphocytes, NK cells rapidly exert cytotoxicity against transformed cells that exhibit decreased MHC-I expression, without antigen priming. RT increases the expression of stress-induced ligands on tumor cells, such as NKG2D ligands, boosting their susceptibility to NK cell-mediated killing [38]. Nevertheless, studies specifically addressing the RT-mediated reprogramming of NK cells remain limited, and further investigation is required to clarify how different doses and fractionation schedules shape NK cell function.

### 3.4. Synergizing with Advanced Immunotherapies: From Adoptive Cell Transfer to Checkpoint Blockade

RT also serves as a powerful preconditioning approach, significantly enhancing the efficacy of adoptive T cell therapies [39]. It achieves this primarily by reprogramming the TME to improve T cell trafficking and infiltration. As mentioned, RT can activate the STING pathway and enhance CAR T-cell infiltration in both irradiated and non-irradiated tumors, resulting in a potent abscopal effect [29]. Additionally, using RT for pre-conditioning increases CCL2 secretion, which then promotes the infiltration of both exogenous CAR-T cells and endogenous T cells into distant tumors [40]. This improved trafficking is also reflected in higher CXCR3 expression on CAR-T cells that were later detected in non-irradiated tumors [41]. By establishing these chemokine gradients and enhancing T-cell infiltration, RT effectively prepares the environment for both engineered CAR-T cells and endogenous T cells that are disinhibited by systemic immune checkpoint blockade.

## 4. The Pro-Tumor Edge: Radiation-Induced Immunosuppression and ICI Resistance

Despite its potential to activate immunity, RT frequently fosters an immunosuppressive TME that shields the tumor from attack and promotes resistance. This process is driven by the paradoxical activation of certain immune pathways and the subsequent accumulation of suppressive immune cell populations. In the era of immunotherapy, this RT-induced suppression is increasingly recognized as one of the drivers causing ICI resistance, as these cellular networks physically and chemically exclude activated T cells from the TME.

### 4.1. Paradoxical Immunosuppressive Role of the cGAS-STING Pathway

As mentioned, RT can enhance anti-tumor immunity by activating the cGAS-STING pathway; however, this activation is complex and may also cause immunosuppression. Following RT, activation of the cGAS-STING pathway increases the production of chemokines, such as CCL2, CCL7, and CCL12, which attract MDSCs [42]. RT also encourages TAMs to polarize toward the tumor-promoting M2 type by increasing IL-34 levels and activating the TBK1-STAT6 signaling pathway. This process may also lead to the death of anti-tumor M1 macrophages [43,44]. Moreover, the STING pathway can directly upregulate Foxp3 transcription, promoting the differentiation of naïve CD4^+^ T cells into immunosuppressive Tregs [45]. This paradoxical STING activation creates a hostile microenvironment that can disable the cytotoxic CD8^+^ T cells activated by ICI treatments.

### 4.2. Accumulation of Suppressive Immune Cell Populations

**Figure 2:**
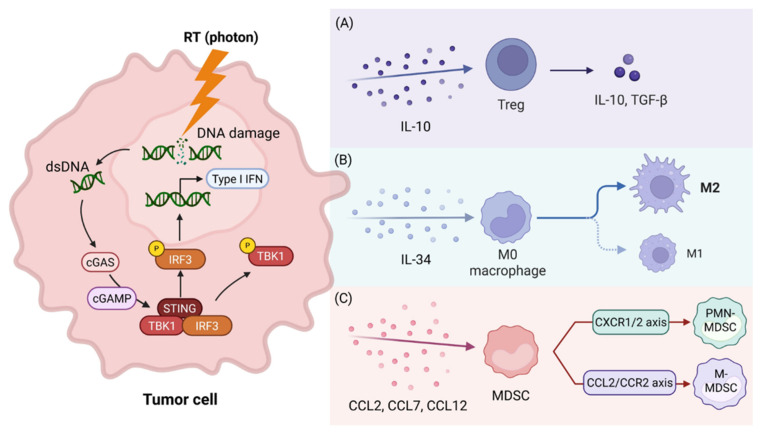
Radiation therapy (RT) causes DNA damage, releasing cytosolic double-stranded DNA (dsDNA) and activating pro-tumor immune responses. Cytoplasmic dsDNA is recognized by the enzyme cGAS, which produces the second messenger cGAMP. cGAMP then binds to STING, activating TBK1. This activation leads to the phosphorylation of IRF3, which is subsequently translocated to the nucleus. Ultimately, this process triggers the production of type I interferon. The activation of the cGAS/STING pathway contributes to the infiltration of pro-tumor immune cells. This can be summarized as follows: (**A**) The cGAS/STING pathway increases the production of IL-10, which promotes the infiltration of Tregs. (**B**) Activation of the cGAS/STING pathway elevates IL-34 levels, leading to TAM polarization towards the M2 phenotype. (**C**) The cGAS/STING pathway enhances the expression of chemokines CCL2, CCL7, and CCL12, which ultimately recruit MDSCs. Specifically, the CXCR1/2 axis recruits PMN-MDSCs, whereas the CCL2/CCR2 axis recruits M-MDSCs. Created in BioRender. Chiang, C.-H. (2026) https://BioRender.com/0apqtx6.

RT not only fosters a favorable TME for immunotherapy but can also create a strongly immunosuppressive TME via two complementary mechanisms, as shown in Figure 2. Attraction and accumulation of key immunosuppressive cells, including Tregs and MDSCs [46], and the production of immunosuppressive and pro-angiogenic factors [47,48]. These factors collectively contribute to immune exclusion, a major phenotype of ICI-resistant tumors.

### 4.3. Regulatory T Cells (Tregs)

The buildup of Tregs acts as a major obstacle to successful cancer treatment [49]. Tregs decrease the cytotoxicity of CD8^+^ T cells and inhibit the differentiation of CD4^+^ T cells, mainly by secreting inhibitory cytokines such as IL-10 and TGF-β [50,51]. Importantly, Tregs are inherently radioresistant [52,53], which poses a significant challenge and particularly hinders treatment outcomes when RT is part of the therapy.

Studies have demonstrated that Tregs are less sensitive to γ-irradiation than conventional CD4^+^ helper T cells [52] and can survive even after receiving a lethal dose of radiation [54]. This resilience is partly due to molecular mechanisms that protect them from cell death. Tregs are known to express the GITR receptor at high levels, which counteract apoptotic signals. RT can further elevate GITR levels in a dose-dependent manner. Additionally, the expression of the survival-promoting protein Akt also increases in Tregs after irradiation, further supporting their resistance [55,56,57,58].

Furthermore, RT-induced TGF-β1 helps recruit Tregs to tumors [59], and the Treg population often increases in a dose-dependent manner after both whole-body and local irradiation [60,61]. Radiation alters Treg function by altering the expression of activation markers and cytokines. For instance, RT regulates Treg activity by triggering IL-10 secretion [54]. Also, RT can activate STAT3 signaling and promote Treg recruitment, proliferation, and subsequent immunosuppression [62].

Because Tregs strongly inhibit the specific effector cells that ICIs are designed to activate, targeting Tregs offers a promising approach to overcoming radioresistance and boosting the effectiveness of combined radio-immunotherapy. Recent research indicates that suppressing Tregs can improve radioresistance in multiple cancer types [23,62]. For instance, combining an anti-GITR antibody with RT has been shown to greatly improve treatment outcomes by reducing Treg levels in a preclinical NSCLC model [63]. It has also been demonstrated that anti-PD-1 therapy causes the accumulation of CD103^+^ Tregs, which suppress CD8^+^ T cell activity and limit overall treatment effectiveness in a glioblastoma model. Incorporating anti-CD25 to target Tregs markedly improves the results of combined RT and anti-PD-1 therapy [64]. This suggests that integrating immunotherapy methods that deplete or inhibit Tregs with RT offers a promising treatment strategy.

### 4.4. M2-Type Macrophages

RT can also promote tumor progression by facilitating the polarization and infiltration of immunosuppressive and pro-tumor M2 macrophages, which further stimulate angiogenesis and release immunosuppressive factors such as IL-10 and TGF-β [65]. Following irradiation, tumor cells and macrophages release signals, including CCL-2, CCL-8, and colony-stimulating factor (CSF). These signals create an environment rich in chemokines that encourages TAM infiltration and their differentiation into the pro-tumor M2 phenotype [66,67]. The prevalence of M2 macrophages in the TME following RT is reinforced by their natural radioresistance compared to anti-tumor M1 cells. This grants them a clear survival benefit post-treatment, particularly in the hypoxic conditions typical of tumor regions [68]. This process is also connected to endothelial-to-mesenchymal transition (EndMT), a cellular change associated with tumor recurrence after RT and immunosuppression. It increases CXCR4 levels, leading to abnormal TAM recruitment and subsequent M2 polarization [69,70]. In ICI therapy, M2 macrophages often serve as a physical sink for checkpoint inhibitors, frequently trapping anti-PD-1 antibodies and blocking their access to the targeted T cells [71,72]. Recently, a study showed that TAMs also express PD-1, which affects their polarization and anti-tumor activity, and that conditioned medium from siPD-1-treated macrophages was found to inhibit tumor cell migration and invasion [73]. Conversely, studies have shown that anti-PD-1 [74] or anti-PD-L1 [75] therapy can convert M2 TAMs back to M1 phenotypes, thereby improving overall efficacy.

### 4.5. Myeloid-Derived Suppressor Cells (MDSCs)

Finally, RT can increase the number of MDSCs, a heterogeneous population of immature myeloid cells that protect tumors by inhibiting T-cell functions. Monocytic MDSCs (M-MDSCs) can differentiate into M2-like TAMs when exposed to signals such as CSF-1, IL-10, or TGF-β [76], which becomes elevated in the tumor microenvironment following RT. Meanwhile, granulocytic MDSCs (PMN-MDSCs) share characteristics with N2-type neutrophils [77]. The RT-triggered cGAS-STING pathway is essential for recruiting MDSCs by activating NF-κB. This activation then promotes CXCR1/2 and CCL2/CCR2 signaling, leading to the recruitment of PMN-MDSCs and M-MDSCs, respectively [78]. The CSF-1/CSF-1R signaling pathway is a crucial mechanism for attracting MDSCs into tumors [79] after RT. By depleting essential amino acids in the microenvironment and producing reactive nitrogen species, the increased MDSC levels effectively inhibit T-cell proliferation and activity, making ICI treatments ineffective [80,81].

## 5. Cancer-Associated Fibroblasts (CAFs): Stromal Mediators of RT-Induced Immunosuppression

Beyond immune cells, cancer-associated fibroblasts (CAFs) are also a major component of the TME. CAFs play a key role in driving tumor progression and establishing escape routes, including metastasis and immune evasion. [82,83]. CAFs enhance radioresistance across various malignancies [84], including lung cancer [85], pancreatic cancer [86], and colorectal cancer [87]. CAFs secrete cytokines, chemokines, and growth factors, including TGF-β and CXCL12, which promote stromal activation, extracellular matrix remodeling, and immune exclusion [87,88]. This molecular crosstalk fosters a complex interaction between CAFs and other infiltrating populations, such as leukocytes and macrophages, thereby shaping an immunosuppressive TME. By continuously secreting collagen and overexpressing extracellular matrix (ECM) components, CAFs construct a dense physical barrier that restricts T-cell infiltration [89]. CAFs can also suppress effector immune cells, including NK cells [90], while simultaneously recruiting immunosuppressive myeloid populations, such as TAMs [85] and MDSCs, via the STAT3–CCL2 axis [91]. In parallel, CAFs may impair CD4^+^ T-cell function and favor the expansion or differentiation of regulatory T cells, further limiting anti-tumor immunity [90].

The radiobiological response of CAFs represents a critical hurdle in RT. Unlike malignant cells that may undergo ICD following irradiation, CAFs exhibit profound radioresistance [91]. Even at high cumulative doses (e.g., 10 fractions of 1.8 Gy, totaling 18 Gy), CAFs isolated from human colorectal cancer evaded cell death and exhibited minimal morphologic changes. Conversely, irradiated CAFs supported colorectal cancer cell growth by secreting IGF1, thereby activating the paracrine IGF1R/Akt/mTOR survival pathway [92]. Crucially for the immunological landscape, irradiated CAFs may also remain immunologically “silent.” Similar observations were made in human non-small cell lung cancer (NSCLC)-derived CAFs exposed to a total dose of 18 Gy (delivered as either a single fraction or 3 × 6 Gy) [93]. Instead of undergoing apoptosis, irradiated CAFs entered a state of senescence. Furthermore, unlike irradiated tumor cells, these senescent CAFs did not exhibit the immunogenic cell death characteristics. They were unable to release DAMPs or elicit type I interferon (IFN-I) responses [93]. These distinction underscores the unique biological responses of CAFs to radiation compared to those of tumor cells. Although RT can kill tumor cells, it also activates residual CAFs, prompting them to support tumor progression.

Beyond immune evasion through this silent phenotype, irradiated CAFs may also contribute to an immunosuppressive microenvironment by impairing the function of infiltrating immune cells. Studies by Inigo Martínez-Zubiaurre and colleagues have extensively demonstrated that CAFs retain their potent immunosuppressive functions even after exposure to clinical doses of radiation [94]. In DCs, CAF-conditioned medium was shown to skew maturing DCs toward a tolerogenic, immature-like phenotype, characterized by reduced expression of activation markers and impaired functional maturation. Notably, this suppressive effect was partially attenuated when CAFs were exposed to fractionated irradiation with 3 × 6 Gy, but not single high-dose irradiation with 1 × 18 Gy [95]. In particular, 1 × 18 Gy CAF-conditioned medium was associated with reduced phosphorylation of NF-κB/p65 at S536, suggesting sustained inhibition of canonical NF-κB activation rather than relief of CAF-mediated DC suppression [95]. On the other hand, RT appears insufficient to rescue NK cells from CAF-mediated suppression. Under basal conditions, CAFs inhibit NK cell proliferation and cytotoxicity, partly associated with reduced degranulation, as indicated by decreased LAMP-1 expression. In contrast to the partial recovery observed in DC-related functions after 3 × 6 Gy irradiation, irradiated CAFs maintain their immunosuppressive ability against NK cells regardless of the radiation dosage strategy. Moreover, irradiated CAFs actively upregulate HLA-E and PVR (CD155) expression, thereby further escaping NK cell-mediated clearance [96]. Together, these findings suggest that the immunomodulatory consequences of CAF irradiation are highly dependent on both RT fractionation and the immune cell population involved. Therefore, irradiated CAFs should not be viewed merely as passive survivors of RT, but as persistent stromal regulators that may shape whether RT promotes immune activation or reinforces local immunosuppression.

## 6. Factors That Tip the Balance: Optimizing RT for Checkpoint Blockade

### 6.1. The Impact of Dose and Fractionation on ICI Synergy

The immunological effects of RT heavily depend on the dose and fractionation schedule, which can determine whether the treatment leads to a pro-inflammatory state that synergizes with ICIs or an immunosuppressive one that causes ICI resistance. Different dosing strategies have unique and sometimes opposing impacts on the tumor microenvironment. High single doses of RT can cause strong ICD and innate immune activation, including DC maturation and type I interferon production. For instance, high-dose RT can trigger the expression of Trex1, an exonuclease that degrades cytoplasmic DNA, thereby preventing STING activation and its downstream immune-priming consequences [97]. In some preclinical models, a single 10 Gy fraction elicited better tumor control and stronger CD8^+^ T-cell infiltration than the same total dose delivered in smaller fraction sizes [98], and a 30 Gy ablative dose can decrease the proportion of MDSCs and the expression of PD-L1 and vascular endothelial growth factor (VEGF) receptor in the CT26 mouse model [99].

However, extremely high doses pose significant challenges for immunotherapy. For example, high single doses promote M2 macrophage polarization, leading to their buildup in chronic hypoxic areas, especially in the high single-dose group [100,101]. Importantly for ICI synergy, a single 20 Gy fraction was much less effective at triggering systemic abscopal responses when combined with anti-CTLA-4 than fractionated regimens in murine breast and colorectal cancer models [102].

Dividing the dose into schemes like 7.5 Gy × 2 or 5 Gy × 3 often enhances anti-tumor immunity while keeping Treg levels low. Hypofractionation is often considered the best partner for ICIs. Preclinical studies show that regimens like 7.5 Gy × 2 provide optimal tumor suppression by increasing tumor-specific IFN-γ^+^ splenocytes while reducing splenic Tregs [60]. Clinically, stereotactic body RT (SBRT), a form of hypofractionation, increases circulating effector and memory T cells. This stands in sharp contrast to conventional, prolonged fractionation (e.g., 2 Gy/day, 5 days per week for weeks), which often leads to severe lymphopenia [103], a strong negative prognostic marker for ICI efficacy, as it physically depletes the very T cells that anti-PD-1 and anti-CTLA-4 drugs rely on to attack the tumor. However, the advantages of fractionation are not universal; some high-dose fractionated schedules may still foster an immunosuppressive environment by increasing M2 macrophage infiltration and polarization [104].

A third distinct strategy involves using low-dose radiotherapy (LDRT, ≤2 Gy per fraction) as a powerful immune adjuvant to reprogram the TME and overcome ICI resistance. Weekly 1 Gy irradiation activates immune pathways, induces chemokines, and recruits NK cells, DCs, and T cells. LDRT has been shown to improve the effectiveness of Treg-targeting immunotherapies by reducing suppressive cells and increasing CD8^+^ infiltration [23], thereby raising the anti-tumor CD8^+^/Foxp3^+^ ratio. Even when lower total RT doses are insufficient on their own, their efficacy can be robustly restored when combined with immunotherapy, often by polarizing macrophages toward the beneficial M1-like phenotype [105]. Optimizing both dose and fractionation is therefore crucial to maximize tumor control while promoting a favorable immune environment for ICIs.

### 6.2. Radiation Type: Preserving the Immune Repertoire for ICIs

The choice of radiation type significantly impacts the reprogramming of the tumor immune microenvironment, with particle therapies like proton and carbon ion radiation often eliciting a more favorable anti-tumor response for subsequent checkpoint blockade than conventional photon therapy [106].

Photon therapy typically exhibits an immunosuppressive profile; it markedly lowers CD4^+^ and CD8^+^ T-cell counts while raising the proportion and proliferation of immunosuppressive Tregs. Additionally, photon therapy mainly elevates IL-6, a cytokine linked to tumor promotion, and reduces the presence of cytotoxic CD8^+^ T cells [107]. By destroying the host’s circulating lymphocyte pool, photon therapy can inadvertently sabotage concurrent ICI administration. In contrast, proton therapy is associated with a more favorable outcome. M1 macrophages are shown to be more resistant to proton irradiation than M2 macrophages [108], and there is no significant infiltration of M2 macrophages or MDSCs after treatment [109]. An advanced technique, flash proton therapy (FPT), appears even more advantageous for ICI synergy, as it has been shown to reduce M2 macrophages and Tregs while increasing M1 infiltration compared to traditional proton therapy [110]. Carbon ion radiotherapy (CIRT) currently offers the most immunologically beneficial profile and stands out as the ideal physical modality to combine with ICIs. CIRT actively inhibits M2 polarization while promoting M1 macrophage infiltration [111], an effect opposite to that observed with photon therapy. Crucially, CIRT significantly elevates pro-inflammatory cytokines such as IFN-γ, IL-2, and IL-1β, enhances the effector function of CD8^+^ T cells [107], and has been shown to decrease both Treg and MDSC populations [112]. Current research shows that photon and proton therapies usually increase Tregs, whereas CIRT largely circumvents this problem and other related immunosuppression issues, such as the induction of TGF-β, IL-6, and VEGF. Consequently, CIRT creates a highly immunogenic TME that is ideally prepared to respond to immune checkpoint blockade [111].

## 7. Dose Limitations of RT: Myelosuppressive Injury and Immune Depletion

Although adjusting the radiation dose and fractionation schemes could improve therapeutic efficacy, their clinical application is limited by a critical challenge: myelosuppression. This injury is caused by damage to hematopoietic stem/progenitor cells (HSPCs), which are highly sensitive to irradiation [113]. For certain malignancies, particularly those requiring irradiation of large bone marrow-rich volumes, RT can severely compromise the hematopoietic compartment and impair immune cell replenishment.

Once HSPCs are compromised, hematopoietic and immune cell replenishment is impaired, while circulating effector lymphocytes may also be rapidly depleted during irradiation. Moreover, in response to acute injury, the hematopoietic system initiates emergency myelopoiesis to quickly restore damaged compartments. However, this accelerated, dysregulated process can promote the expansion and release of immature MDSCs with strong immunosuppressive capacity. Once recruited into the TME, these MDSCs profoundly suppress T-cell function and weaken anti-tumor immunity [114]. Consequently, the resulting systemic lymphopenia is strongly correlated with poor patient prognosis and diminished overall survival [115].

## 8. Future Perspectives and Therapeutic Strategies

The future of RT is closely linked to immunotherapy. The main clinical goal is to develop treatment strategies that harness the ability of RT to trigger anti-tumor immune responses while simultaneously minimizing its potential to promote tumor growth and resistance. Currently, this goal is being addressed through various combination approaches focused on overcoming resistance to ICIs.

### 8.1. Optimizing Cellular and Immune Combinations

The core strategy utilizes RT to activate the TME and boost PD-L1 expression, making immunologically “cold” tumors more responsive to T-cell-based therapies like anti-PD-1/PD-L1 and anti-CTLA-4. To further enhance this effect, RT is also used increasingly as a preparatory step for adoptive T cell treatments, such as CAR-T cell therapy. In this role, RT upregulates antigen presentation and establishes a favorable chemokine gradient, significantly enhancing the infiltration and efficacy of engineered T cells. Furthermore, because the success of these T cell-centric therapies is frequently hindered by localized immunosuppression, combining RT with myeloid-targeting agents that target CSF-1R [116], CCR2 [117], or CXCR4 [118]. It offers a powerful strategy to directly combat resistance by blocking the recruitment and function of pro-tumor MDSCs and M2 macrophages.

### 8.2. The Metabolic Roadblock: Dampening Innate Immunity

Although these cellular-level strategies are highly promising, tumors’ inherent metabolic adaptations often weaken their ability to maintain a strong anti-tumor response, especially when mediated by the cGAS-STING pathway. Following irradiation, cancer cells heavily rely on upregulated metabolic networks to mitigate ROS and repair DNA double-strand breaks. This metabolic plasticity not only facilitates radioresistance [119] but also limits the accumulation of cytosolic DNA fragments, thereby prematurely dampening cGAS-STING signaling. Consequently, fully unlocking the immunogenic potential of RT requires a strategy that goes beyond inflicting initial DNA damage to actively disabling the tumor’s metabolic escape routes.

### 8.3. Broadening the Metabolic Axis: Glucose, Amino Acids, and Regulated Cell Death

This insight has led to a paradigm shift centered on the link between innate immunity and tumor metabolism. TME is a fiercely competitive space that often influences immunotherapy outcomes. Tumors quickly deplete glucose through the Warburg effect and also consume essential amino acids, resulting in immune cell starvation and creating an acidic, immunosuppressive microenvironment [120]. For instance, Tumor-driven glucose depletion and elevated lactate levels directly hinder the glycolytic capacity of effector CD8^+^ T cells. This nutritional deprivation pushes T cells into metabolic exhaustion, making PD-1/PD-L1 blockade largely ineffective because the T cells lack the energy to attack even when “unleashed” [121].

Furthermore, the dysregulation of amino acid metabolism poses a severe immunological barrier. The depletion of tryptophan by indoleamine 2,3-dioxygenase (IDO) and the consumption of arginine by RT-recruited MDSCs generate metabolites like kynurenine that actively suppress T-cell proliferation and drive Treg differentiation [121]. The combination of an IDO inhibitor with RT and ICIs could improve survival and tumor control rates in a preclinical glioblastoma model [122].

Beyond nutrient competition, RT intrinsically alters tumor metabolism by inducing ferroptosis, an iron-dependent form of regulated cell death driven by excessive lipid peroxidation [123]. Crucially, this metabolic cell death directly intersects with adaptive immunity. ICI-activated CD8^+^ T cells secrete IFN-γ, which downregulates tumor antioxidant defenses, further sensitizing cancer cells to RT-induced ferroptosis [124] and creating a mutually reinforcing loop of tumor clearance. However, the immunological consequences of ferroptosis are highly context-dependent, as ferroptosis may promote tumor cell killing while simultaneously compromising anti-tumor T-cell fitness. Lipid metabolic stress also contributes to CD8^+^ T cell dysfunction within the TME. High CD36 expression in tumor-infiltrating CD8^+^ T cells has been associated with tumor progression and poor survival. CD36 mediates fatty acid uptake, particularly the accumulation of polyunsaturated fatty acids (PUFAs), leading to lipid peroxidation and ferroptosis in CD8^+^ T cells. This process suppresses cytotoxic cytokine production and impairs anti-tumor function, which can be reversed by blocking CD36 or inhibiting ferroptosis [125].

To effectively utilize this unique metabolic regulation, future strategies should combine RT with metabolic modulators that inhibit glycolysis, IDO, or arginase to restore nutrient availability in the TME. Ferroptosis-related metabolic pathways may provide an additional opportunity to enhance the efficacy and safety of RT. Recently, cholesterol has been shown to suppress ferroptosis by increasing the levels of antioxidant metabolites CoQ10 and squalene. Therefore, blocking CoQ and squalene synthesis simultaneously may enhance RT-induced ferroptosis and improve treatment efficacy [126]. However, given the dual role of ferroptosis in both tumor control and normal tissue injury, these interventions must be optimized for different cell types. For example, RT-induced myelosuppressive injury may also be mitigated through metabolic modulation. Exogenous serine supplementation has been reported to protect HSPCs from ferroptosis. This effect was linked to enhanced mitochondrial serine catabolism, which helped preserve the hematopoietic compartment during RT-induced myelosuppression [127]. Similarly, excess cholesterol prevents HSPCs from ferroptosis, thereby protecting them from RT-induced myelosuppression [128]. Collectively, these findings suggest that metabolic targeting should aim to enhance ferroptotic susceptibility in tumor cells while preserving immune-cell fitness and hematopoietic integrity. By therapeutically “starving” the tumor and simultaneously “re-arming” the immune system metabolically, these strategies prevent early suppression of RT-triggered innate immunity. This combined immunometabolic tactic effectively dismantles the immunosuppressive environment, turning radioresistant, nutrient-starved “cold” tumors into metabolically active, “hot” tumors that respond well to checkpoint blockade.

## 9. Conclusions

Ultimately, gaining a more detailed mechanistic understanding of how RT induces immune and metabolic reprogramming will facilitate true personalization of radio-immunotherapy. By fine-tuning RT parameters and selecting the right immunotherapeutic and metabolic partners tailored to a patient’s unique tumor environment, RT can evolve from a mere cytotoxic treatment to a vital cornerstone of modern cancer immunotherapy, particularly when combined with ICIs.

## Data Availability

No new data were created or analyzed in this study. Data sharing is not applicable to this article.

## References

[1] Dertinger H., Jung H. (1970). Direct and indirect action of radiation. Molecular Radiation Biology: The Action of Ionizing Radiation on Elementary Biological Objects.

[2] Azzam E.I., Little J.B. (2004). The radiation-induced bystander effect: Evidence and significance. Hum. Exp. Toxicol..

[3] Grass G.D., Krishna N., Kim S. (2016). The immune mechanisms of abscopal effect in radiation therapy. Curr. Probl. Cancer.

[4] Kodet O., Němejcova K., Strnadová K., Havlínová A., Dundr P., Krajsová I., Štork J., Smetana K., Lacina L. (2021). The Abscopal Effect in the Era of Checkpoint Inhibitors. Int. J. Mol. Sci..

[5] Pitt J.M., Kroemer G., Zitvogel L. (2017). Immunogenic and Non-immunogenic Cell Death in the Tumor Microenvironment. Adv. Exp. Med. Biol..

[6] Liu J., Hong M., Li Y., Chen D., Wu Y., Hu Y. (2022). Programmed Cell Death Tunes Tumor Immunity. Front. Immunol..

[7] Green D.R., Droin N., Pinkoski M. (2003). Activation-induced cell death in T cells. Immunol. Rev..

[8] Obeid M., Tesniere A., Ghiringhelli F., Fimia G.M., Apetoh L., Perfettini J.-L., Castedo M., Mignot G., Panaretakis T., Casares N. (2007). Calreticulin exposure dictates the immunogenicity of cancer cell death. Nat. Med..

[9] Verma A., Arora A., Bhatt A.N., Arya M.B., Prasad A.K., Parmar V.S., Dwarakanath B.S. (2022). Radiosensitization of calreticulin-overexpressing human glioma cell line by the polyphenolic acetate 7, 8-diacetoxy-4-methylcoumarin. Cancer Rep..

[10] Alvaro T., de la Cruz-Merino L., Henao-Carrasco F., Villar Rodríguez J.L., Vicente Baz D., Codes Manuel de Villena M., Provencio M. (2010). Tumor microenvironment and immune effects of antineoplastic therapy in lymphoproliferative syndromes. J. Biomed. Biotechnol..

[11] Gong Z., Jia Q., Guo J., Li C., Xu S., Jin Z., Chu H., Wan Y.Y., Zhu B., Zhou Y. (2023). Caspase-8 contributes to an immuno-hot microenvironment by promoting phagocytosis via an ecto-calreticulin-dependent mechanism. Exp. Hematol. Oncol..

[12] Yu Y., Feng S., Wei S., Zhong Y., Yi G., Chen H., Liang L., Chen H., Lu X. (2019). Extracellular ATP activates P2X7R-NF-κB (p65) pathway to promote the maturation of bone marrow-derived dendritic cells of mice. Cytokine.

[13] Murshid A., Gong J., Calderwood S.K. (2012). The role of heat shock proteins in antigen cross presentation. Front. Immunol..

[14] Li J.G., Du Y.M., Yan Z.D., Yan J., Zhuansun Y.X., Chen R., Zhang W., Feng S.L., Ran P.X. (2016). CD80 and CD86 knockdown in dendritic cells regulates Th1/Th2 cytokine production in asthmatic mice. Exp. Ther. Med..

[15] Mackenzie K.J., Carroll P., Martin C.-A., Murina O., Fluteau A., Simpson D.J., Olova N., Sutcliffe H., Rainger J.K., Leitch A. (2017). cGAS surveillance of micronuclei links genome instability to innate immunity. Nature.

[16] Li G., Zhao X., Zheng Z., Zhang H., Wu Y., Shen Y., Chen Q. (2024). cGAS-STING pathway mediates activation of dendritic cell sensing of immunogenic tumors. Cell Mol. Life Sci..

[17] Dovedi S.J., Adlard A.L., Lipowska-Bhalla G., McKenna C., Jones S., Cheadle E.J., Stratford I.J., Poon E., Morrow M., Stewart R. (2014). Acquired resistance to fractionated radiotherapy can be overcome by concurrent PD-L1 blockade. Cancer Res..

[18] Herter-Sprie G.S., Koyama S., Korideck H., Hai J., Deng J., Li Y.Y., Buczkowski K.A., Grant A.K., Ullas S., Rhee K. (2016). Synergy of radiotherapy and PD-1 blockade in Kras-mutant lung cancer. JCI Insight.

[19] Antonia S.J., Villegas A., Daniel D., Vicente D., Murakami S., Hui R., Yokoi T., Chiappori A., Lee K.H., de Wit M. (2017). Durvalumab after Chemoradiotherapy in Stage III Non-Small-Cell Lung Cancer. N. Engl. J. Med..

[20] Reits E.A., Hodge J.W., Herberts C.A., Groothuis T.A., Chakraborty M., K.Wansley E., Camphausen K., Luiten R.M., de Ru A.H., Neijssen J. (2006). Radiation modulates the peptide repertoire, enhances MHC class I expression, and induces successful antitumor immunotherapy. J. Exp. Med..

[21] Tailor A., Estephan H., Parker R., Woodhouse I., Abdulghani M., Nicastri A., Jones K., Salatino S., Muschel R., Humphrey T. (2022). Ionizing Radiation Drives Key Regulators of Antigen Presentation and a Global Expansion of the Immunopeptidome. Mol. Cell Proteom..

[22] Lhuillier C., Rudqvist N.P., Yamazaki T., Zhang T., Charpentier M., Galluzzi L., Dephoure N., Clement C.C., Santambrogio L., Zhou X.K. (2021). Radiotherapy-exposed CD8+ and CD4+ neoantigens enhance tumor control. J. Clin. Investig..

[23] Ji D., Song C., Li Y., Xia J., Wu Y., Jia J., Cui X., Yu S., Gu J. (2020). Combination of radiotherapy and suppression of Tregs enhances abscopal antitumor effect and inhibits metastasis in rectal cancer. J. Immunother. Cancer.

[24] Beauford S.S., Kumari A., Garnett-Benson C. (2020). Ionizing radiation modulates the phenotype and function of human CD4+ induced regulatory T cells. BMC Immunol..

[25] Zeng X., Jin X., Leng J., Zhang S., Wang Y., Chen J., Zhang S., Teng L., Hu Z., Zhou S. (2025). High-dose radiation induces dendritic cells maturation by promoting immunogenic cell death in nasopharyngeal carcinoma. Front. Immunol..

[26] Telarovic I., Yong C.S.M., Kurz L., Vetrugno I., Reichl S., Fernandez A.S., Cheng H.-W., Winkler R., Guckenberger M., Kipar A. (2024). Delayed tumor-draining lymph node irradiation preserves the efficacy of combined radiotherapy and immune checkpoint blockade in models of metastatic disease. Nat. Commun..

[27] Zhang Y., Hu H.-H., Zhou S.-H., Xia W.-Y., Zhang Y., Zhang J.-P., Fu X.-L., Yu W. (2023). PET-based radiomics visualizes tumor-infiltrating CD8 T cell exhaustion to optimize radiotherapy/immunotherapy combination in mouse models of lung cancer. Biomark. Res..

[28] Zhou S., Zhu M., Wei X., Mu P., Shen L., Wang Y., Wan J., Zhang H., Xia F., Zhang Z. (2024). Low-dose radiotherapy synergizes with iRGD-antiCD3-modified T cells by facilitating T cell infiltration. Radiother. Oncol..

[29] Kostopoulos N., Costabile F., Krimitza E., Beghi S., Goia D., Perales-Linares R., Thyfronitis G., LaRiviere M.J., Chong E.A., Schuster S.J. (2024). Local radiation enhances systemic CAR T-cell efficacy by augmenting antigen crosspresentation and T-cell infiltration. Blood Adv..

[30] Oba T., Long M.D., Keler T., Marsh H.C., Minderman H., Abrams S.I., Liu S., Ito F. (2020). Overcoming primary and acquired resistance to anti-PD-L1 therapy by induction and activation of tumor-residing cDC1s. Nat. Commun..

[31] Herrera F.G., Ronet C., Ochoa de Olza M., Barras D., Crespo I., Andreatta M., Corria-Osorio J., Spill A., Benedetti F., Genolet R. (2022). Low-Dose Radiotherapy Reverses Tumor Immune Desertification and Resistance to Immunotherapy. Cancer Discov..

[32] Dehne N., Mora J., Namgaladze D., Weigert A., Brüne B. (2017). Cancer cell and macrophage cross-talk in the tumor microenvironment. Curr. Opin. Pharmacol..

[33] Chen Y., Song Y., Du W., Gong L., Chang H., Zou Z. (2019). Tumor-associated macrophages: An accomplice in solid tumor progression. J. Biomed. Sci..

[34] Teresa Pinto A., Laranjeiro Pinto M., Patrícia Cardoso A., Monteiro C., Teixeira Pinto M., Filipe Maia A., Castro P., Figueira R., Monteiro A., Marques M. (2016). Ionizing radiation modulates human macrophages towards a pro-inflammatory phenotype preserving their pro-invasive and pro-angiogenic capacities. Sci. Rep..

[35] Chan H.-W., Sheung P.-W., Tsao S.T.-M., Wu C.-Y., Chuang H.-Y. (2025). Gold nanoparticle-loaded macrophages enhance radiotherapy via immune remodeling in oral cancer. Mater. Today Biol..

[36] Cheng F., Lienlaf M., Wang H.W., Perez-Villarroel P., Lee C., Woan K., Rock-Klotz J., Sahakian E., Woods D., Pinilla-Ibarz J. (2014). A novel role for histone deacetylase 6 in the regulation of the tolerogenic STAT3/IL-10 pathway in APCs. J. Immunol..

[37] Noonepalle S.K.R., Grindrod S., Aghdam N., Li X., Gracia-Hernandez M., Zevallos-Delgado C., Jung M., Villagra A., Dritschilo A. (2023). Radiotherapy-induced Immune Response Enhanced by Selective HDAC6 Inhibition. Mol. Cancer Ther..

[38] Lin X., Liu Z., Dong X., Wang K., Sun Y., Zhang H., Wang F., Chen Y., Ling J., Guo Y. (2024). Radiotherapy enhances the anti-tumor effect of CAR-NK cells for hepatocellular carcinoma. J. Transl. Med..

[39] Obertopp N., Bekker R.A., Grass G.D., Zelenka T., Thomas A., Potez M., Ali J., Blauvelt J., Hall A.M., Hall M.S. (2025). Local Single-Dose Radiation Improves Adoptive Cell Therapy with Tumor-Infiltrating Lymphocytes. Int. J. Radiat. Oncol. Biol. Phys..

[40] Ma X., Zhang W., Zeng M., Asavasupreechar T., Kang S., Li Y., Yu L. (2024). Systemic tumor regression with synergy therapy: Radiotherapy and CAR-T. Cell Death Discov..

[41] Quach H.T., Skovgard M.S., Villena-Vargas J., Bellis R.Y., Chintala N.K., Amador-Molina A., Bai Y., Banerjee S., Saini J., Xiong Y. (2023). Tumor-Targeted Nonablative Radiation Promotes Solid Tumor CAR T-cell Therapy Efficacy. Cancer Immunol. Res..

[42] Ostrand-Rosenberg S., Horn L.A., Ciavattone N.G. (2019). Radiotherapy Both Promotes and Inhibits Myeloid-Derived Suppressor Cell Function: Novel Strategies for Preventing the Tumor-Protective Effects of Radiotherapy. Front. Oncol..

[43] Jiang Q., Chen Z., Jiang J., Chen Q., Lan H., Zhu J., Mao W. (2025). The role of cGAS-STING in remodeling the tumor immune microenvironment induced by radiotherapy. Crit. Rev. Oncol. Hematol..

[44] Ma R., Ji T., Chen D., Dong W., Zhang H., Yin X., Ma J., Liang X., Zhang Y., Shen G. (2016). Tumor cell-derived microparticles polarize M2 tumor-associated macrophages for tumor progression. Oncoimmunology.

[45] Ni H., Zhang H., Li L., Huang H., Guo H., Zhang L., Li C., Xu J.X., Nie C.P., Li K. (2022). T cell-intrinsic STING signaling promotes regulatory T cell induction and immunosuppression by upregulating FOXP3 transcription in cervical cancer. J. Immunother. Cancer.

[46] Boustani J., Lecoester B., Baude J., Latour C., Limagne E., Ladjohoulou R., Morgand V., Froidurot L., Ghiringhelli F., Truc G. (2024). Targeting two radiation-induced immunosuppressive pathways to improve the efficacy of normofractionated radiation therapy in a preclinical colorectal cancer model. Int. J. Radiat. Biol..

[47] Zhu H., Zhang S. (2018). Hypoxia inducible factor-1α/vascular endothelial growth factor signaling activation correlates with response to radiotherapy and its inhibition reduces hypoxia-induced angiogenesis in lung cancer. J. Cell Biochem..

[48] Lan Y., Moustafa M., Knoll M., Xu C., Furkel J., Lazorchak A., Yeung T.L., Hasheminasab S.M., Jenkins M.H., Meister S. (2021). Simultaneous targeting of TGF-β/PD-L1 synergizes with radiotherapy by reprogramming the tumor microenvironment to overcome immune evasion. Cancer Cell.

[49] Song D., Ding Y. (2023). A new target of radiotherapy combined with immunotherapy: Regulatory T cells. Front. Immunol..

[50] Pan Y., Zhou H., Sun Z., Zhu Y., Zhang Z., Han J., Liu Y., Wang Q. (2025). Regulatory T cells in solid tumor immunotherapy: Effect, mechanism and clinical application. Cell Death Dis..

[51] Wang Y., Ma Y., Fang Y., Wu S., Liu L., Fu D., Shen X. (2012). Regulatory T cell: A protection for tumour cells. J. Cell Mol. Med..

[52] Qu Y., Jin S., Zhang A., Zhang B., Shi X., Wang J., Zhao Y. (2010). Gamma-Ray Resistance of Regulatory CD4 CD25 Foxp3 T Cells in Mice. Radiat. Res..

[53] Baba J., Watanabe S., Saida Y., Tanaka T., Miyabayashi T., Koshio J., Ichikawa K., Nozaki K., Koya T., Deguchi K. (2012). Depletion of radio-resistant regulatory T cells enhances antitumor immunity during recovery from lymphopenia. Blood.

[54] Liu S., Sun X., Luo J., Zhu H., Yang X., Guo Q., Song Y., Sun X. (2015). Effects of radiation on T regulatory cells in normal states and cancer: Mechanisms and clinical implications. Am. J. Cancer Res..

[55] Cao M., Cabrera R., Xu Y., Liu C., Nelson D. (2009). Gamma irradiation alters the phenotype and function of CD4^+^CD25^+^ regulatory T cells. Cell Biol. Int..

[56] Wang M., Gou X., Wang L. (2012). Protein kinase B promotes radiation-induced regulatory T cell survival in bladder carcinoma. Scand. J. Immunol..

[57] Li C.G., He M.R., Wu F.L., Li Y.J., Sun A.M. (2013). Akt promotes irradiation-induced regulatory T-cell survival in hepatocellular carcinoma. Am. J. Med. Sci..

[58] Wu C.T., Hsieh C.C., Yen T.C., Chen W.C., Chen M.F. (2015). TGF-β1 mediates the radiation response of prostate cancer. J. Mol. Med..

[59] Kachikwu E.L., Iwamoto K.S., Liao Y.P., DeMarco J.J., Agazaryan N., Economou J.S., McBride W.H., Schaue D. (2011). Radiation enhances regulatory T cell representation. Int. J. Radiat. Oncol. Biol. Phys..

[60] Schaue D., Ratikan J.A., Iwamoto K.S., McBride W.H. (2012). Maximizing tumor immunity with fractionated radiation. Int. J. Radiat. Oncol. Biol. Phys..

[61] Guo S., Yao Y., Tang Y., Xin Z., Wu D., Ni C., Huang J., Wei Q., Zhang T. (2023). Radiation-induced tumor immune microenvironments and potential targets for combination therapy. Signal Transduct. Target. Ther..

[62] Oweida A.J., Darragh L., Phan A., Binder D., Bhatia S., Mueller A., Court B.V., Milner D., Raben D., Woessner R. (2019). STAT3 Modulation of Regulatory T Cells in Response to Radiation Therapy in Head and Neck Cancer. J. Natl. Cancer Inst..

[63] Schoenhals J.E., Cushman T.R., Barsoumian H.B., Li A., Cadena A.P., Niknam S., Younes A.I., Caetano M.D.S., Cortez M.A., Welsh J.W. (2018). Anti-glucocorticoid-induced Tumor Necrosis Factor-Related Protein (GITR) Therapy Overcomes Radiation-Induced Treg Immunosuppression and Drives Abscopal Effects. Front. Immunol..

[64] van Hooren L., Handgraaf S.M., Kloosterman D.J., Karimi E., van Mil L.W.H.G., Gassama A.A., Solsona B.G., de Groot M.H.P., Brandsma D., Quail D.F. (2023). CD103^+^ regulatory T cells underlie resistance to radio-immunotherapy and impair CD8^+^ T cell activation in glioblastoma. Nat. Cancer.

[65] Biswas S.K., Mantovani A. (2010). Macrophage plasticity and interaction with lymphocyte subsets: Cancer as a paradigm. Nat. Immunol..

[66] Becherini C., Lancia A., Detti B., Lucidi S., Scartoni D., Ingrosso G., Carnevale M.G., Roghi M., Bertini N., Orsatti C. (2023). Modulation of tumor-associated macrophage activity with radiation therapy: A systematic review. Strahlenther. Onkol..

[67] Yang H., Lei Z., He J., Zhang L., Lai T., Zhou L., Wang N., Tang Z., Sui J., Wu Y. (2024). Single-cell RNA sequencing reveals recruitment of the M2-like CCL8(high) macrophages in Lewis lung carcinoma-bearing mice following hypofractionated radiotherapy. J. Transl. Med..

[68] Leblond M.M., Peres E.A., Helaine C., Gerault A.N., Moulin D., Anfray C., Divoux D., Petit E., Bernaudin M., Valable S. (2017). M2 macrophages are more resistant than M1 macrophages following radiation therapy in the context of glioblastoma. Oncotarget.

[69] Bischoff J. (2019). Endothelial-to-Mesenchymal Transition. Circ. Res..

[70] Choi S.H., Kim A.R., Nam J.K., Kim J.M., Kim J.Y., Seo H.R., Lee H.J., Cho J., Lee Y.J. (2018). Tumour-vasculature development via endothelial-to-mesenchymal transition after radiotherapy controls CD44v6(+) cancer cell and macrophage polarization. Nat. Commun..

[71] Van Dam S., Krijgsman D., Küçükköse E., Verdonschot M.E.L., Amini M., Blokx W.A.M., Van Eijs M.J.M., Verheijden R.J., Kranenburg O., Suijkerbuijk K.P.M. (2025). Anti-PD-1 treatment response is associated with the influx of circulating myeloid and T-cell subsets into the metastatic melanoma tumor microenvironment. Br. J. Cancer.

[72] Peranzoni E., Lemoine J., Vimeux L., Feuillet V., Barrin S., Kantari-Mimoun C., Bercovici N., Guérin M., Biton J., Ouakrim H. (2018). Macrophages impede CD8 T cells from reaching tumor cells and limit the efficacy of anti–PD-1 treatment. Proc. Natl. Acad. Sci. USA.

[73] Jiang H., Pang J., Li T., Akofala A., Zhou X., Yi C., Ning S., Gao X., Qiao Y., Kou J. (2025). PD-1 regulates the anti-tumor immune function of macrophages through JAK2-STAT3 signaling pathway in colorectal cancer tumor microenvironment. J. Transl. Med..

[74] Dhupkar P., Gordon N., Stewart J., Kleinerman E.S. (2018). Anti-PD-1 therapy redirects macrophages from an M2 to an M1 phenotype inducing regression of OS lung metastases. Cancer Med..

[75] Xiong H., Mittman S., Rodriguez R., Moskalenko M., Pacheco-Sanchez P., Yang Y., Nickles D., Cubas R. (2019). Anti–PD-L1 Treatment Results in Functional Remodeling of the Macrophage Compartment. Cancer Res..

[76] Tcyganov E., Mastio J., Chen E., Gabrilovich D.I. (2018). Plasticity of myeloid-derived suppressor cells in cancer. Curr. Opin. Immunol..

[77] Zhou J., Nefedova Y., Lei A., Gabrilovich D. (2018). Neutrophils and PMN-MDSC: Their biological role and interaction with stromal cells. Semin. Immunol..

[78] Li K., Shi H., Zhang B., Ou X., Ma Q., Chen Y., Shu P., Li D., Wang Y. (2021). Myeloid-derived suppressor cells as immunosuppressive regulators and therapeutic targets in cancer. Signal Transduct. Target. Ther..

[79] Trikha P., Carson W.E. (2014). Signaling pathways involved in MDSC regulation. Biochim. Biophys. Acta.

[80] Zhang C., Wang H., Aji T., Li Z., Li Y., Ainiwaer A., Rousu Z., Li J., Wang M., Deng B. (2024). Targeting myeloid-derived suppressor cells promotes antiparasitic T-cell immunity and enhances the efficacy of PD-1 blockade. Nat. Commun..

[81] Weber R., Fleming V., Hu X., Nagibin V., Groth C., Altevogt P., Utikal J., Umansky V. (2018). Myeloid-Derived Suppressor Cells Hinder the Anti-Cancer Activity of Immune Checkpoint Inhibitors. Front. Immunol..

[82] Kay E.J., Zanivan S. (2025). The tumor microenvironment is an ecosystem sustained by metabolic interactions. Cell Rep..

[83] Monteran L., Erez N. (2019). The Dark Side of Fibroblasts: Cancer-Associated Fibroblasts as Mediators of Immunosuppression in the Tumor Microenvironment. Front. Immunol..

[84] Wang Z., Tang Y., Tan Y., Wei Q., Yu W. (2019). Cancer-associated fibroblasts in radiotherapy: Challenges and new opportunities. Cell Commun. Signal.

[85] Wang Y., Gan G., Wang B., Wu J., Cao Y., Zhu D., Xu Y., Wang X., Han H., Li X. (2017). Cancer-associated Fibroblasts Promote Irradiated Cancer Cell Recovery Through Autophagy. EBioMedicine.

[86] Mantoni T.S., Lunardi S., Al-Assar O., Masamune A., Brunner T.B. (2011). Pancreatic stellate cells radioprotect pancreatic cancer cells through beta1-integrin signaling. Cancer Res..

[87] Liu L., Zhang Z., Zhou L., Hu L., Yin C., Qing D., Huang S., Cai X., Chen Y. (2020). Cancer associated fibroblasts-derived exosomes contribute to radioresistance through promoting colorectal cancer stem cells phenotype. Exp. Cell Res..

[88] Barker H.E., Paget J.T., Khan A.A., Harrington K.J. (2015). The tumour microenvironment after radiotherapy: Mechanisms of resistance and recurrence. Nat. Rev. Cancer.

[89] Masuda H. (2025). Cancer-associated fibroblasts in cancer drug resistance and cancer progression: A review. Cell Death Discov..

[90] Elyada E., Bolisetty M., Laise P., Flynn W.F., Courtois E.T., Burkhart R.A., Teinor J.A., Belleau P., Biffi G., Lucito M.S. (2019). Cross-Species Single-Cell Analysis of Pancreatic Ductal Adenocarcinoma Reveals Antigen-Presenting Cancer-Associated Fibroblasts. Cancer Discov..

[91] De P., Aske J., Dey N. (2021). Cancer-Associated Fibroblast Functions as a Road-Block in Cancer Therapy. Cancers.

[92] Tommelein J., De Vlieghere E., Verset L., Melsens E., Leenders J., Descamps B., Debucquoy A., Vanhove C., Pauwels P., Gespach C.P. (2018). Radiotherapy-Activated Cancer-Associated Fibroblasts Promote Tumor Progression through Paracrine IGF1R Activation. Cancer Res..

[93] Berzaghi R., Gundersen K., Dille Pedersen B., Utne A., Yang N., Hellevik T., Martinez-Zubiaurre I. (2024). Immunological signatures from irradiated cancer-associated fibroblasts. Front. Immunol..

[94] Gorchs L., Hellevik T., Bruun J.A., Camilio K.A., Al-Saad S., Stuge T.B., Martinez-Zubiaurre I. (2015). Cancer-associated fibroblasts from lung tumors maintain their immunosuppressive abilities after high-dose irradiation. Front. Oncol..

[95] Berzaghi R., Tornaas S., Lode K., Hellevik T., Martinez-Zubiaurre I. (2021). Ionizing Radiation Curtails Immunosuppressive Effects From Cancer-Associated Fibroblasts on Dendritic Cells. Front. Immunol..

[96] Yang N., Lode K., Berzaghi R., Islam A., Martinez-Zubiaurre I., Hellevik T. (2020). Irradiated Tumor Fibroblasts Avoid Immune Recognition and Retain Immunosuppressive Functions Over Natural Killer Cells. Front. Immunol..

[97] Vanpouille-Box C., Alard A., Aryankalayil M.J., Sarfraz Y., Diamond J.M., Schneider R.J., Inghirami G., Coleman C.N., Formenti S.C., Demaria S. (2017). DNA exonuclease Trex1 regulates radiotherapy-induced tumour immunogenicity. Nat. Commun..

[98] Cocito C., Branchtein M., Zhou X.K., Gongora T., Dahmane N., Greenfield J.P. (2025). Single-dose radiotherapy is more effective than fractionation when combined with anti-PD-1 immunotherapy in glioblastoma. Sci. Rep..

[99] Lan J., Li R., Yin L.M., Deng L., Gui J., Chen B.Q., Zhou L., Meng M.B., Huang Q.R., Mo X.M. (2018). Targeting Myeloid-derived Suppressor Cells and Programmed Death Ligand 1 Confers Therapeutic Advantage of Ablative Hypofractionated Radiation Therapy Compared with Conventional Fractionated Radiation Therapy. Int. J. Radiat. Oncol. Biol. Phys..

[100] Wijerathne H., Langston J.C., Yang Q., Sun S., Miyamoto C., Kilpatrick L.E., Kiani M.F. (2021). Mechanisms of radiation-induced endothelium damage: Emerging models and technologies. Radiother. Oncol..

[101] Chen F.H., Chiang C.S., Wang C.C., Tsai C.S., Jung S.M., Lee C.C., McBride W.H., Hong J.H. (2009). Radiotherapy decreases vascular density and causes hypoxia with macrophage aggregation in TRAMP-C1 prostate tumors. Clin. Cancer Res..

[102] Dewan M.Z., Galloway A.E., Kawashima N., Dewyngaert J.K., Babb J.S., Formenti S.C., Demaria S. (2009). Fractionated but not single-dose radiotherapy induces an immune-mediated abscopal effect when combined with anti-CTLA-4 antibody. Clin. Cancer Res..

[103] Palermo B., Bottero M., Panetta M., Faiella A., Sperduti I., Masi S., Frisullo G., Foddai M.L., Cordone I., Nisticò P. (2023). Stereotactic Ablative Radiation Therapy in 3 Fractions Induces a Favorable Systemic Immune Cell Profiling in Prostate Cancer Patients. Oncoimmunology.

[104] Sheng Y., Zhang B., Xing B., Liu Z., Chang Y., Wu G., Zhao Y. (2023). Cancer-Associated Fibroblasts Exposed to High-Dose Ionizing Radiation Promote M2 Polarization of Macrophages, Which Induce Radiosensitivity in Cervical Cancer. Cancers.

[105] Laurent P.A., Shi L., Bouarroudj L., Benzazon N., Gerbé De Thoré M., Liu W., Aglave M., Bergeron P., Naulin F., Sitterlé L. (2025). Low-dose radiotherapy enhances the efficacy of PD-L1 blockade and induces the abscopal effect. J. Immunother. Cancer.

[106] Ma L. (2022). From Photon Beam to Accelerated Particle Beam: Antimetastasis Effect of Combining Radiotherapy with Immunotherapy. Front. Public Health.

[107] Spina C.S., Tsuruoka C., Mao W., Sunaoshi M.M., Chaimowitz M., Shang Y., Welch D., Wang Y.F., Venturini N., Kakinuma S. (2021). Differential Immune Modulation with Carbon-Ion Versus Photon Therapy. Int. J. Radiat. Oncol. Biol. Phys..

[108] Genard G., Wera A.-C., Huart C., Le Calve B., Penninckx S., Fattaccioli A., Tabarrant T., Demazy C., Ninane N., Heuskin A.-C. (2018). Proton irradiation orchestrates macrophage reprogramming through NFκB signaling. Cell Death Dis..

[109] Mirjolet C., Nicol A., Limagne E., Mura C., Richard C., Morgand V., Rousseau M., Boidot R., Ghiringhelli F., Noel G. (2021). Impact of proton therapy on antitumor immune response. Sci. Rep..

[110] Zhang A., Fan L., Liu Q., Zuo X., Zhu J. (2025). Immunological Effects of Proton Radiotherapy: New Opportunities and Challenges in Cancer Therapy. Cancer Innov..

[111] Chiblak S., Tang Z., Lemke D., Knoll M., Dokic I., Warta R., Moustafa M., Mier W., Brons S., Rapp C. (2019). Carbon irradiation overcomes glioma radioresistance by eradicating stem cells and forming an antiangiogenic and immunopermissive niche. JCI Insight.

[112] Zhou H., Yang P., Li H., Zhang L., Li J., Zhang T., Sheng C., Wang J. (2021). Carbon ion radiotherapy boosts anti-tumour immune responses by inhibiting myeloid-derived suppressor cells in melanoma-bearing mice. Cell Death Discov..

[113] Zhang Y., Chen X., Wang X., Chen J., Du C., Wang J., Liao W. (2024). Insights into ionizing radiation-induced bone marrow hematopoietic stem cell injury. Stem Cell Res. Ther..

[114] Swann J.W., Olson O.C., Passegue E. (2024). Made to order: Emergency myelopoiesis and demand-adapted innate immune cell production. Nat. Rev. Immunol..

[115] Kleinberg L., Sloan L., Grossman S., Lim M. (2019). Radiotherapy, Lymphopenia, and Host Immune Capacity in Glioblastoma: A Potentially Actionable Toxicity Associated with Reduced Efficacy of Radiotherapy. Neurosurgery.

[116] Stafford J.H., Hirai T., Deng L., Chernikova S.B., Urata K., West B.L., Brown J.M. (2016). Colony stimulating factor 1 receptor inhibition delays recurrence of glioblastoma after radiation by altering myeloid cell recruitment and polarization. Neuro-Oncology.

[117] Wang J., Saung M.T., Li K., Fu J., Fujiwara K., Niu N., Muth S., Wang J., Xu Y., Rozich N. (2022). CCR2/CCR5 inhibitor permits the radiation-induced effector T cell infiltration in pancreatic adenocarcinoma. J. Exp. Med..

[118] Lecavalier-Barsoum M., Chaudary N., Han K., Pintilie M., Hill R.P., Milosevic M. (2019). Targeting CXCL12/CXCR4 and myeloid cells to improve the therapeutic ratio in patient-derived cervical cancer models treated with radio-chemotherapy. Br. J. Cancer.

[119] Nguyen L., Dobiasch S., Schneider G., Schmid R.M., Azimzadeh O., Kanev K., Buschmann D., Pfaffl M.W., Bartzsch S., Schmid T.E. (2021). Impact of DNA repair and reactive oxygen species levels on radioresistance in pancreatic cancer. Radiother. Oncol..

[120] Ricci J.-E. (2025). Tumor-induced metabolic immunosuppression: Mechanisms and therapeutic targets. Cell Rep..

[121] Zhang R., Mao G., Tang Y., Li C., Gao Y., Nie W., Song T., Liu S., Zhang P., Tao K. (2024). Inhibition of glycolysis enhances the efficacy of immunotherapy via PDK-mediated upregulation of PD-L1. Cancer Immunol. Immunother..

[122] Ladomersky E., Zhai L., Lenzen A., Lauing K.L., Qian J., Scholtens D.M., Gritsina G., Sun X., Liu Y., Yu F. (2018). IDO1 Inhibition Synergizes with Radiation and PD-1 Blockade to Durably Increase Survival Against Advanced Glioblastoma. Clin. Cancer Res..

[123] Lang X., Green M.D., Wang W., Yu J., Choi J.E., Jiang L., Liao P., Zhou J., Zhang Q., Dow A. (2019). Radiotherapy and Immunotherapy Promote Tumoral Lipid Oxidation and Ferroptosis via Synergistic Repression of SLC7A11. Cancer Discov..

[124] Wang W., Green M., Choi J.E., Gijón M., Kennedy P.D., Johnson J.K., Liao P., Lang X., Kryczek I., Sell A. (2019). CD8+ T cells regulate tumour ferroptosis during cancer immunotherapy. Nature.

[125] Ma X., Xiao L., Liu L., Ye L., Su P., Bi E., Wang Q., Yang M., Qian J., Yi Q. (2021). CD36-mediated ferroptosis dampens intratumoral CD8(+) T cell effector function and impairs their antitumor ability. Cell Metab..

[126] Sun Q., Liu D., Cui W., Cheng H., Huang L., Zhang R., Gu J., Liu S., Zhuang X., Lu Y. (2023). Cholesterol mediated ferroptosis suppression reveals essential roles of Coenzyme Q and squalene. Commun. Biol..

[127] Du C., Liu C., Yu K., Zhang S., Fu Z., Chen X., Liao W., Chen J., Zhang Y., Wang X. (2024). Mitochondrial serine catabolism safeguards maintenance of the hematopoietic stem cell pool in homeostasis and injury. Cell Stem Cell.

[128] Liu C., Liao W., Chen J., Yu K., Wu Y., Zhang S., Chen M., Chen F., Wang S., Cheng T. (2023). Cholesterol confers ferroptosis resistance onto myeloid-biased hematopoietic stem cells and prevents irradiation-induced myelosuppression. Redox Biol..

